# Association of *HK2* and *NCK2* with Normal Tension Glaucoma in the Japanese Population

**DOI:** 10.1371/journal.pone.0054115

**Published:** 2013-01-22

**Authors:** Dong Shi, Tomoyo Funayama, Yukihiko Mashima, Yoshimasa Takano, Ai Shimizu, Kotaro Yamamoto, MinGe Mengkegale, Akiko Miyazawa, Noriko Yasuda, Takeo Fukuchi, Haruki Abe, Hidenao Ideta, Kohji Nishida, Toru Nakazawa, Julia E. Richards, Nobuo Fuse

**Affiliations:** 1 Department of Ophthalmology, Tohoku University Graduate School of Medicine, Sendai, Miyagi, Japan; 2 Department of Ophthalmology, the Fourth Affiliated Hospital, China Medical University, Shenyang, Liaoning, China; 3 Department of Chemistry, Faculty of Education, Bunkyo University, Koshigaya, Saitama, Japan; 4 Department of Ophthalmology, Keio University School of Medicine, Shinjuku-ku, Tokyo, Japan; 5 Department of Ophthalmology, Tokyo Metropolitan Police Hospital, Tokyo, Japan; 6 Division of Ophthalmology and Visual Science, Graduate School of Medical and Dental Sciences, Niigata University, Niigata, Japan; 7 Ideta Eye Hospital, Kumamoto, Japan; 8 Department of Ophthalmology, Osaka University Graduate School of Medicine, Suita, Osaka, Japan; 9 Department of Ophthalmology and Visual Sciences, W. K. Kellogg Eye Center, University of Michigan, Ann Arbor, Michigan, United States of America; 10 Department of Epidemiology, University of Michigan, Ann Arbor, Michigan, United States of America; 11 Department of Integrative Genomics, Tohoku Medical Megabank Organization, Sendai, Miyagi, Japan; University of Missouri-Columbia, United States of America

## Abstract

Although family studies and genome-wide association studies have shown that genetic factors play a role in glaucoma, it has been difficult to identify the specific genetic variants involved. We tested 669 single nucleotide polymorphisms (SNPs) from the region of chromosome 2 that includes the GLC1B glaucoma locus for association with primary open-angle glaucoma (POAG) and normal tension glaucoma (NTG) in the Japanese population. We performed a two-stage case-control study. The first cohort consisted of 123 POAG cases, 121 NTG cases and 120 controls: the second cohort consisted of 187 POAG cases, 286 NTG cases, and 271 controls. Out of six SNPs showing significant association with POAG in the first round screening, seven SNPs were tested in the second round. Rs678350 in the *HK2* gene coding sequence showed significant allelic (p = 0.0027 in Stage Two, 2.7XE-4 in meta-analysis) association with POAG, and significant allelic (p = 4.7XE-4 in Stage Two, 1.0XE-5 in meta-analysis) association with NTG. Although alleles in the *TMEM182* gene did not show significant association with glaucoma in the second round, subjects with the A/A allele in *TMEM182* rs869833 showed worse visual field mean deviation (p = 0.01). Even though rs2033008 in the *NCK2* gene coding sequence did not show significant association in the first round, it had previously shown association with NTG so it was tested for association with NTG in round 2 (p = 0.0053 in Stage Two). Immunohistochemistry showed that both *HK2* and *NCK2* are expressed in the retinal ganglion cell layer. Once multi-testing was taken into account, only *HK2* showed significant association with POAG and NTG in Stage Two. Our data also support previous reports of *NCK2* association with NTG, and raise questions about what role *TMEM182* might play in phenotypic variability. Our data suggest that *HK2* may play an important role in NTG in the Japanese population.

## Introduction

Glaucoma is a complex, heterogeneous disease characterized by a progressive degeneration of the optic nerve fibers, and is the second highest cause of blindness worldwide affecting approximately 70 million people [1]. The most common type of open-angle glaucoma, primary open-angle glaucoma (POAG), is associated with elevated intraocular pressure (IOP) [2], and another less-common subgroup of open-angle glaucoma, called low-tension glaucoma (LTG) [3], [4] or normal tension glaucoma (NTG) is associated with IOP that does not rise outside of the normal range [5]. The prevalence of NTG is reported to be higher among the Japanese than among Caucasians [6], [7]. This is an important medical and public health problem because simple screening programs based on detection of elevated IOP are not effective in a population where NTG is highly prevalent. Thus, an accurate diagnostic test for presymptomatic detection of individuals at risk for glaucoma, especially NTG in Japan, is urgently needed.

Open-angle glaucoma is a genetically heterogeneous disorder attributed to the interaction of multiple genes and environmental factors [8], [9]. More than 15 POAG loci have been identified by linkage, and five open-angle glaucoma genes located within those loci have been identified [10], [11]. More recently, genome-wide association studies (GWAS) using high-density single nucleotide polymorphism (SNP) arrays have been used to identify genetic risk factors involved in the common, complex forms of open-angle glaucoma that do not show classical Mendelian inheritance patterns. Burdon et al. identified susceptibility loci at TMCO1 and CDKN2B-AS1 that contribute to severe forms of glaucoma [12]. Ramdas et al. used meta-analysis of data from six separate studies to find significant evidence that three common variants of CDKN2B, ATOH7 and SIX1 are associated with POAG [13]. Wiggs et al. found significant evidence that genetic variants in CDKN2B-AS1 and a gene desert on 8q22 are associated with optic nerve damage in glaucoma [14].

Based on a linkage study involving 6 Caucasian families in the UK, the *GLC1B* locus for adult-onset open-angle glaucoma was identified at chromosome 2cen–q13 [15]. The patients in these families had clinical characteristics of low to moderate IOP, disease onset in their late 40 s, and a good response to medical therapy, and those phenotypes mimic the majority of Japanese NTG cases. Thus, the screening of the gene around GLC1B locus may be useful for diagnosis of POAG and NTG in the general Japanese population.

The purpose of this study was to screen for candidate genes for POAG and NTG on chromosome 2, around the GLC1B (glaucoma 1, open angle, B) locus in unrelated Japanese patients, using high density SNP scanning and case-control association. Here we report one gene that shows significant association with POAG and NTG, support for a previously reported association with NTG, and a gene for which genotype is predictive of severity of mean deviation on the visual field test.

## Results

### A Two-stage Case-control Study of SNPs on Chromosome2

**Figure 1:**
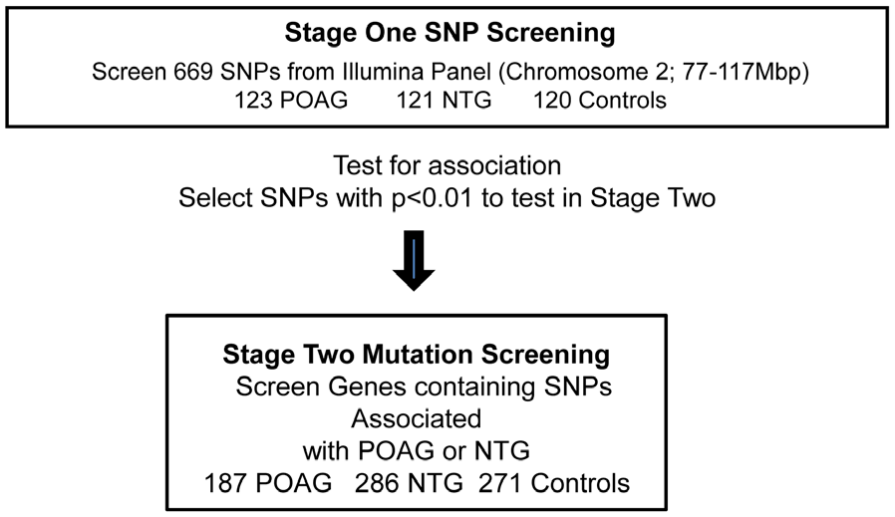
Experimental Study Design. The study used a first stage of SNP screening in one population to identify SNPs and genes to be tested in a second population through SNP association testing and mutation screening of genes containing SNPs associated with glaucoma. Stage Two tested SNPs for association in a second population and did mutation screening in that second population in genes containing SNPs that showed significant association with POAG or NTG (p<0.01) in Stage One of our study or that had been previously reported to show significant evidence of association [16].

To identify a gene associated with glaucoma we did a high-density scan of the region around GLC1B on chromosome 2 by screening 669 SNPs on chromosome 2 in a two-stage case-control study design (Figure 1). We were especially interested in whether any SNPs that fall within genes in the GLC1B region might be associated with POAG or NTG (Table 1). Among genes from this region we found fourteen SNPs that show significant evidence of association with POAG, and nine SNPs that show significant evidence of association with NTG. Four of the SNP alleles which show significant evidence of association are identical between the POAG and NTG subjects.

**Table 1:** Stage one Test of SNPs in GLC1B-Region Genes for Association with POAG or NTG.

Genomic Information			POAG			NTG			Control
rs number	Location	Gene Symbol	MAF	Odds ratio (CI)	p value*	MAF	Odds ratio (CI)	p value*	MAF
rs741788	2p13	*DCTN1*	0.455	1.47 (1.02–2.12)	0.038	0.455	1.47 (1.02–2.11)	0.040	0.363
rs909177		*DCTN1*	0.455	1.44 (1.00–2.08)	0.047	0.463	1.49 (1.03–2.14)	0.032	0.367
rs740277		*DCTN1*	0.455	1.44 (1.00–2.08)	0.047	0.463	1.49 (1.03–2.14)	0.032	0.367
rs678350	2p13	*HK2*	0.333	1.50 (1.01–2.23)	0.043	0.371	1.77 (1.19–2.62)	0.004	0.250
rs651071		*HK2*	0.199	0.60 (0.40–0.92)	0.018	0.256	0.84 (0.56–1.25)	0.383	0.292
rs1807090		*HK2*	0.268	1.59 (1.03–2.44)	0.034	0.238	1.35 (0.87–2.10)	0.181	0.188
rs1239066	2p12		0.293	3.04 (1.95–4.72)	0.009	0.269	1.55 (1.01–2.38)	0.045	0.192
rs53915	2p12-p11.1	*CTNNA2*	0.199	0.64 (0.42–0.98)	0.039	0.260	0.91 (0.61–1.36)	0.641	0.279
rs1529385	2p11.2	*LOC129293 (C2orf89)*	0.053	0.39 (0.20–0.77)	0.005	0.087	0.66 (0.37–1.20)	0.173	0.125
rs1053561	2p11.2	*TGOLN2*	0.114	0.53 (0.32–0.88)	0.012	0.178	0.89 (0.56–1.40)	0.609	0.196
rs1562322	2p11.2	*LOC51255 (RNF181)*	0.321	0.88 (0.61–1.29)	0.520	0.256	0.64 (0.43–0.95)	0.027	0.349
rs3024831	2p12-p11.2	*SFTPB*	0.250	0.92 (0.61–1.38)	0.675	0.190	0.65 (0.42–0.99)	0.045	0.267
rs6875	2q11.2	*RW1 (TMEM131)*	0.008	0.21 (0.05–0.98)	0.030	0.025	0.66 (0.23–1.88)	0.431	0.038
rs1982336		*RW1 (TMEM131)*	0.008	0.23 (0.05–1.05)	0.027	0.029	0.75 (0.28–2.05)	0.576	0.038
rs718159		*RW1 (TMEM131)*	0.008	0.21 (0.05–0.98)	0.028	0.029	0.76 (0.28–2.07)	0.587	0.038
rs222	2q11.2	*INPP4A*	0.183	0.69 (0.44–1.06)	0.091	0.161	0.59 (0.38–0.93)	0.021	0.246
rs1530028	2q11.2	*FLJ45273 (LONRF2)*	0.228	0.66 (0.44–0.99)	0.045	0.306	0.99 (0.67–1.46)	0.952	0.308
rs1030902	2q11.2	*ALS2*	0.225	0.65 (0.44–0.98)	0.039	0.314	1.03 (0.70–1.51)	0.892	0.308
rs1369482	2q11.2	*NPAS2*	0.244	0.65 (0.43–0.96)	0.030	0.298	0.85 (0.58–1.24)	0.398	0.333
rs871656	2q12	*IL1R1*	0.337	0.70 (0.49–1.01)	0.058	0.322	0.66 (0.45–0.95)	0.025	0.421
rs878539	2q11.2	*SLC9A2 (NHE2)*	0.463	1.52 (1.06–2.18)	0.024	0.422	1.28 (0.89–1.85)	0.185	0.363
rs869833	2q12.1	*TMEM182*	0.467	1.66 (1.15–2.40)	0.006	0.376	1.14 (0.79–1.65)	0.490	0.346
rs960011		*TMEM182*	0.415	0.57 (0.40–0.82)	0.001	0.512	0.85 (0.60–1.21)	0.232	0.554**
rs2033008	2q12	*NCK2*	0.293	0.76 (0.51–1.11)	0.147	0.252	0.62 (0.42–0.91)	0.015	0.354
rs1027003	2q12		0.110	3.16 (1.46–6.88)	0.002	0.058	1.58 (0.67–3.71)	0.295	0.038
rs1474220	2q12.3	*GCC2*	0.106	0.59 (0.35–1.00)	0.050	0.136	0.79 (0.48–1.30)	0.353	0.167
rs899259	2q13	*EDAR*	0.098	0.66 (0.38–1.14)	0.134	0.075	0.49 (0.28–0.90)	0.019	0.142
rs1509414	2q13	*BENE(MALL)*	0.037	0.40 (0.18–0.88)	0.020	0.041	0.45 (0.21–0.98)	0.039	0.096
rs1567366	2q13	*NPHP1*	0.561***	1.34 (0.94–1.92)	0.105	0.576***	1.44 (1.01–2.07)	0.045	0.488
rs2119112	2q14.2	*MARCO*	0.110	0.57 (0.34–0.95)	0.029	0.107	0.55 (0.33–0.93)	0.025	0.179

* chi-square test.

** minor allele frequency in stage 2 control was 0.494.

*** minor allele in control was major allele in POAG and NTG subjects.

MAF; minor allele frequency, CI; confidence interval.

**Figure 2:**
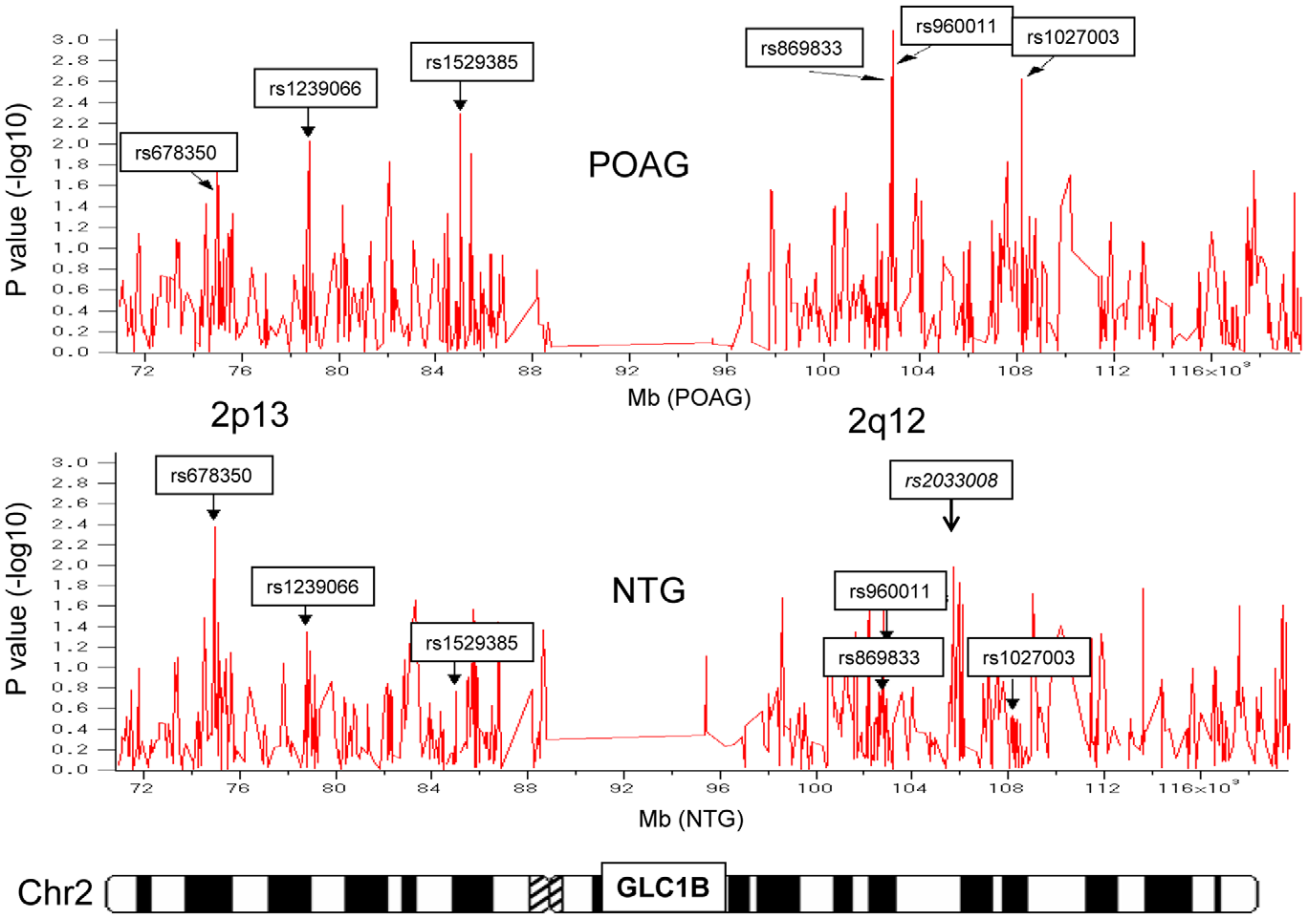
High-density scan of the GLC1B region on chromosome 2 to identify candidate glaucoma genes. Six SNPs that showed significant evidence of association with POAG or NTG (p<0.01) and the previously-reported candidate *NCK2* gene are shown. Vertical line shows p value (−log10), and horizontal line shows chromosomal location (kb).

We identified six SNPs in Stage One that showed evidence of association with POAG (rs1239066, rs1529385, rs869833, rs960011, rs1027003) and two SNPs (rs678350, rs2033008) that showed association with NTG (Figure 2 and Table 2). The SNPs rs869833 and rs960011 are located within the *TMEM182* gene, which contains 5 exons and 229 amino acids. The amino acid sequence of *TMEM182* predicts an evolutionarily-conserved novel transmembrane protein, which consists of four putative membrane-spanning regions indicative of an integral membrane topology. The SNP rs678350 is located within the *Hexokinase 2 (HK2*) gene which contains 18 exons and 917 amino acids. The SNP rs678350 exists on intron1 of *HK2* gene. The *HK2* gene produces a protein product localizes on the outer membrane of mitochondria and plays an important role in intracellular glucose metabolism by catalyzing the conversion of glucose to glucose-6-phosphate. There are no known genes closely neighboring the SNP rs1239066 and rs1027003. We have checked the SNPs near rs1529385 within LOC129293 and TMSB10 and did not find get any positive polymorphisms.

**Table 2:** Stage One and Stage Two Association Test Results.

Stage One Screening								
		POAG			NTG			CNTL
SNP	Minor Allele	MAF	Odds ratio	p value*	MAF	Odds ratio	p value*	MAF
rs1239066	C	0.293	3.04 (1.95–4.72)	0.009	0.269	1.55 (1.01–2.38)	0.045	0.192
rs1529385	T	0.053	0.39 (0.20–0.77)	0.005	0.087	0.66 (0.37–1.20)	0.173	0.125
rs869833	G	0.467	1.66 (1.15–2.40)	0.006	0.376	1.14 (0.79–1.65)	0.490	0.346
rs960011	T	0.415	0.57 (0.40–0.82)	0.001	0.512	0.85 (0.60–1.21)	0.232	0.554
rs1027003	G	0.110	3.16 (1.46–6.88)	0.002	0.058	1.58 (0.67–3.71)	0.295	0.038
rs678350	G	0.333	1.50 (1.01–2.23)	0.043	0.371	1.77 (1.19–2.62)	0.004	0.250
rs2033008	A	0.293	0.76 (0.51–1.11)	0.147	0.252	0.62 (0.42–0.91)	0.015	0.354

Stage Two Screening								
		POAG			NTG			CNTL
SNP	Minor Allele	MAF	Odds ratio	p value*	MAF	Odds ratio	p value*	MAF
rs1239066	C	0.246	1.70 (1.09–2.65)	0.019	0.240	1.64 (1.06–2.51)	0.024	0.161
rs1529385	T	0.122	1.42 (0.73–2.77)	0.305	0.072	0.79 (0.37–1.68)	0.543	0.089
rs869833	G	0.409	0.98 (0.75–1.28)	0.855	0.421	1.03 (0.81–1.30)	0.834	0.415
rs960011	T	0.497	1.13 (0.86–1.47)	0.363	0.481	1.06 (0.84–1.34)	0.641	0.467
rs1027003	G	0.049	1.10 (0.42–2.90)	0.840	0.047	1.04 (0.30–2.83)	0.944	0.045
rs678350	G	0.334	1.58 (1.18–2.11)	0.0027	0.337	1.60 (1.23–2.08)	4.7XE-4	0.242
rs2033008	A	0.297	0.87 (0.65–1.16)	0.348	0.250	0.69 (0.53–0.89)	0.0053	0.327

Meta-analysis								
		POAG			NTG			CNTL
SNP	Minor Allele	MAF	Odds ratio	p value*	MAF	Odds ratio	p value*	MAF
rs1239066	C	0.264	1.65 (1.22–2.23)	0.001	0.248	1.52 (1.14–2.04)	0.005	0.178
rs1529385	T	0.082	0.73 (0.46–1.16)	0.20	0.081	0.71 (0.45–1.14)	0.16	0.109
rs869833	G	0.432	1.17 (0.95–1.45)	0.16	0.408	1.06 (0.87–1.30)	0.54	0.393
rs960011	T	0.465	0.89 (0.72–1.10)	0.28	0.490	0.99 (0.81–1.20)	0.92	0.494
rs1027003	G	0.084	2.17 (1.20–3.92)	0.010	0.053	1.32 (0.69–2.53)	0.42	0.041
rs678350	G	0.334	1.56 (1.23–1.96)	2.7XE-4	0.347	1.65 (1.32–2.05)	1.0XE-5	0.244
rs2033008	A	0.295	0.83 (0.66–1.04)	0.12	0.251	0.66 (0.53–0.82)	2.2XE-4	0.335

MAF; Minor allele frequency, CNTL; Control.

* Fisher's exact test.

We selected three genes for second stage mutation screening. *TMEM182* and *HK2* genes were selected because they contain SNPs that showed significant evidence of association in Stage One in this study. Even though SNPs in the *NCK2* gene showed significant evidence of association (p = 0.014), the *NCK2* gene was selected for second stage mutation screening based of the previous report that it is associated with NTG [16].

The SNP rs1239066 showed significant evidence of association in both Stage One and Stage Two screenings. In the meta-analysis it showed significant evidence of association (POAG, NTG; P = 0.001, 0.005), as did rs1027003 (POAG; P = 0,010) (Table 2).

### *HK2* Variants Detected in this Study

The SNP rs678350 in the *HK2* gene coding sequence showed significant allelic (p = 0.0027 in Stage Two, 2.7XE-4 in meta-analysis) association with POAG, and significant allelic (p = 4.7XE-4 in Stage Two, 1.0XE-5 in meta-analysis) association with NTG (Table 2). The rs678350 showed also a significant difference in genotype frequency (p = 0.0046 and 0.0039) in the POAG and NTG groups (Table 3). In the second round, we screened the *HK2* coding sequence and intron-exon boundaries for mutations in POAG and NTG patients. After direct sequencing, we found 2 coding SNPs; p.Gln142His (A/T at the third nucleotide; rs2229621) in exon 4 and p.Arg844Lys (G/A at the second nucleotide; rs2229629) in exon 17. The allelic frequency of the p.Gln142His (A/T) variant was significantly higher in the NTG group than in the control group (p = 0.025), but it was not higher in the POAG group than in the control group (p = 0.181). The genotype frequency of the p.Gln142His (A/T) variant (dominant model) was significantly higher in the NTG group than in the control group (p = 0.019) but we did not find evidence that the frequency in the POAG group was different from the control group (p = 0.179). There was no evidence of a significant difference between POAG and NTG for p.Arg844Lys. No other mutation was found. We tested the LD block and found no linkage disequilibrium between SNPs rs678350 and rs2229621 (D′ = 0.08). We tested the correlation between the phenotypes POAG or NTG and the genotypes screened in the second stage screening of HK2, and found no association with any phenotypes including age at diagnosis, maximum IOP under medication, and MD value of the visual field (Table 4). None of the polymorphisms showed deviation from Hardy–Weinberg equilibrium (*P*<0.05).

**Table 3:** Stage Two *HK2* SNPs Allele Frequencies in Japanese POAG, NTG and Control Subjects.

	Allele frequency				Genotype			
rs678350	A	G	Odds ratio (CI)	p value*	A/A	A/G	G/G	p value*
POAG	0.666	0.334	1.58 (1.18–2.11)	0.0027	82/187	85/187	20/187	0.0046
NTG	0.663	0.337	1.60 (1.23–2.08)	4.7XE-4	133/286	113/286	40/286	0.0039
Control	0.758	0.242			161/271	89/271	21/271	

	Allele frequency				Genotype			
rs2229621/Q142H	A	T	Odds ratio (CI)	p value*	A/A	A/T	T/T	p value*
POAG	0.738	0.262	1.24 (0.92–1.69)	0.181	102/187	72/187	13/187	0.179
NTG	0.719	0.281	1.36 (1.04–1.79)	0.025	146/286	119/286	21/286	0.019
Control	0.777	0.223			165/271	91/271	15/271	

* Fisher's exact test; dominant model.

G/G or T/T is mutant homozygote, A/G or A/T is heterozygote, and A/A is wild homozygote.

**Table 4:** Correlation between the POAG or NTG Endophenotypes and *HK2* SNPs Screened in Stage Two.

Endophenotype	Age at diagnosis (y.o.)				Maximum IOP* (mmHg)				MD value of the visual field (dB)			
rs678350 genotype	A/A	A/G	G/G	p value**	A/A	A/G	G/G	p value**	A/A	A/G	G/G	p value**
POAG	61.6	55.3	59.0	0.83	24.0	23.6	21.9	0.56	−14.82	−15.64	−11.94	0.53
NTG	57.5	54.9	56.9	0.99	17.1	16.0	17.6	0.64	−10.72	−12.10	−7.14	0.37

rs2229621 genotype	A/A	A/T	T/T	p value**	A/A	A/T	T/T	p value**	A/A	A/T	T/T	p value**
POAG	58.5	58.0	55.5	0.93	24.4	22.9	25.5	0.94	−16.42	−11.94	−17.65	0.65
NTG	57.8	55.2	57.4	0.91	16.1	17.5	16.9	0.63	−11.93	−10.34	−11.16	0.95

* IOP; intraocular pressure (under medication).

** Dunnett's test.

G/G or T/T is mutant homozygote, A/G or A/T is heterozygote, and A/A is wild homozygote.

### *NCK2* Variants Detected in this Study

The SNP rs2033008 in the *NCK2* gene showed a significant difference in allelic frequency (p = 0.015 in Stage One, p = 0.0053 in Stage Two, and 2.2XE-4 in meta-analysis) between controls and NTG, but not between control and POAG status (p = 0.147 in Stage One, 0.35 in Stage Two, 0.12 in meta-analysis) (Table 2 and Table 5). The odds ratio for association with NTG supports a model in which *NCK2* is associated with NTG; OR = 0.69 (0.53–0.89), but the odds ratio for association with POAG was not significant; 0.87 (0.65–1.16) in Stage Two. Only this rs2033008 polymorphism in Stage One showed deviation from Hardy–Weinberg equilibrium (p = 0.030).

**Table 5:** Stage Two *NCK2* SNP rs2033008 Allele Frequencies in Japanese POAG, NTG and Controls Subjects.

	Allele frequency				Genotype			
	T	A	Odds ratio (CI)	p value*	T/T	T/A	A/A	p value**
POAG	0.703	0.297	0.87 (0.65–1.16)	0.35	89/187	85/187	13/187	0.069
NTG	0.750	0.250	0.69 (0.53–0.89)	0.0053	159/286	111/286	16/286	0.0056
Control	0.673	0.327			130/271	105/271	36/271	

* Fisher's exact test,

** Chi-square test.

A/A is mutant homozygote, T/A is heterozygote, and T/T is wild homozygote.

We screened the sequence of the *NCK2* coding sequence and intron-exon boundaries for mutations in POAG and NTG patients and found 1 synonymous coding base change: Thr14Thr (ACC>ACT) in one NTG subject. Although SNP rs2033008 showed significant association but the Thr14Thr variant showed no statistical difference in allele frequency between NTG and normal subjects (p = 0.33). The Thr14Thr heterozygotes (A/A) in the NTG subjects have the worse Mean Deviation value of the visual field compared with human reference sequence (T/T) (p = 0.05) (Table 6).

**Table 6:** Correlation between the POAG or NTG Endophenotypes and *NCK2* SNP rs2033008 Screened in Stage Two.

Endophenotype	Age at diagnosis (y.o.)				Maximum IOP* (mmHg)				MD value of the visual field (dB)			
Genotype	T/T	T/A	A/A	p value**	T/T	T/A	A/A	p value**	T/T	T/A	A/A	p value**
POAG	55.8	61.0	54.2	0.94	23.7	24.2	25.0	0.87	−15.38	−14.94	−12.28	0.63
NTG	56.8	56.0	52.2	0.70	16.7	17.0	16.0	0.79	−9.83	−10.95	−16.28	0.05

* IOP; intraocular pressure (under medication).

** Dunnett's test.

A/A is mutant homozygote, T/A is heterozygote, and T/T is wild homozygote.

The single NTG subject with the Thr14Thr variant was a 50 year old woman whose father also had NTG. Her age at diagnosis was 40 years old. Her initial IOP was 15 mmHg in each eye. The mean deviation (MD) of the visual field test was −3.87 dB in the right eye, and −1.97 dB in the left eye, reflecting a mild NTG phenotype.

### *TMEM182* Variants Detected in this Study

We found no mutations in *TMEM182* coding sequence and intron-exon boundaries, for mutations in POAG and NTG patients. After finding association for SNPs rs869833 and rs960011 in the POAG and NTG subjects in Stage One, we did not find this association confirmed in Stage Two SNP testing (Table 7). When we tested for correlation between Stage Two case endophenotypes and TMEM182 genotype, we found association with the MD value of the visual field in POAG subjects. The POAG subjects homozygous for the A/A allele of SNP rs869833 have worse Mean Deviation value of the visual field test compared with those who carry the G/G genotype in Stage Two subjects (p = 0.01) (Table 8).

**Table 7:** Stage Two *TMEM182* SNP Allele Frequencies in Japanese POAG, NTG and Control Subjects.

	Allele frequency			Genotype			
rs869833	A	G	p value*	A/A	A/G	G/G	p value**
POAG	0.590	0.410	0.855	61/187	99/187	27/187	0.095
NTG	0.579	0.421	0.834	94/286	143/286	49/286	0.269
Control	0.585	0.415		100/271	117/271	54/271	

	Allele frequency			Genotype			
rs960011	C	T	p value*	C/C	C/T	T/T	p value**
POAG	0.503	0.497	0.363	45/187	98/187	44/187	0.479
NTG	0.520	0.480	0.641	76/286	145/286	65/286	0.782
Control	0.533	0.467		79/271	131/271	61/271	

* Fisher's exact test,

** Chi-square test.

G/G or T/T is mutant homozygote, A/G or C/T is heterozygote, and A/A or C/C is wild homozygote.

**Table 8:** Correlation between the POAG or NTG Endophenotypes and *TMEM182* SNPs Screened in Stage Two.

Endophenotype	Age at diagnosis (y.o.)				Maximum IOP* (mmHg)				MD value of the visual field (dB)			
rs869833 Genotype	A/A	A/G	G/G	p value**	A/A	A/G	G/G	p value**	A/A	A/G	G/G	p value**
POAG	54.8	61.1	56.0	0.95	24.7	23.8	23.3	0.80	−17.5	−15.0	−9.95	0.01
NTG	55.8	55.7	59.9	0.40	16.9	16.9	16.4	0.84	−12.60	−9.62	−12.21	0.98

rs960011Genotype	C/C	C/T	T/T	p value**	C/C	C/T	T/T	p value**	C/C	C/T	T/T	p value**
POAG	55.5	57.3	62.0	0.29	24.8	24.4	23.2	0.73	−14.2	−18.5	−9.44	0.37
NTG	53.8	56.4	60.1	0.14	17.1	17.3	15.7	0.16	−10.77	−11.02	−11.76	0.88

* IOP; intra ocular pressure (under medication),

** Dunnett's test.

G/G or T/T is mutant homozygote, A/G or C/T is heterozygote, and A/A or C/C is wild homozygote.

### Immunohistochemistory of the *HK2* and *NCK2*

**Figure 3:**
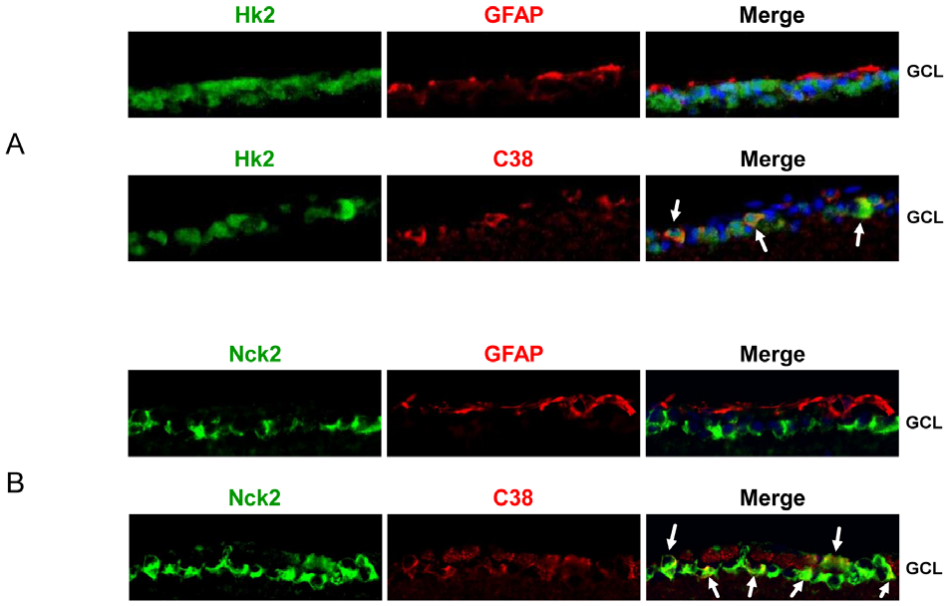
*Hk2* and *Nck2* Immunohistochemistory. Representative IHC photographs showing Hk2, Nck2, astrocyte maker (GFAP) and RGC marker (C38) in the retinas of untreated mice. Arrows indicated the co-localization area.

Representative immunohistochemistory (IHC) photographs with *Hk2, Nck2*, astrocyte marker (GFAP) and retinal ganglion cell marker (C38) on the retinas of untreated mice were shown (Fig. 3, A, B). *Hk2* and *Nck2* were strongly immunoreactive in the ganglion cell layer. C38 signals co-localized with *Hk2* or *Nck2* in the ganglion cell layer, as indicated by arrows (Fig. 3 A, B). *Hk2* expression is only located in the ganglion cell layer. *Nck* is expressed in the ganglion cell layer, inner nuclear layer, and outer plexiform layer, with the highest level of expression in the ganglion cell layer.

## Discussion

### *HK2* in POAG and NTG subjects

The rs678350 in the *HK2* gene coding sequence showed significant allelic (p = 0.043 in Stage One, p = 0.0027 in Stage Two, 2.7XE-4 in meta-analysis association with POAG, and significant allelic (p = 0.004 in Stage One, p = 4.7XE-4 in Stage Two, 1.0XE-5 in meta-analysis) association with NTG (Table 2). The rs678350 polymorphism showed a significant case-control difference in genotype frequency (*p* = 0.0046 and 0.0039) in the POAG and NTG groups (Table 3). However, there was no association of this SNP with glaucoma endophenotypes including age at diagnosis, maximum intra ocular pressure under medication, and MD value of the visual field. So the *HK2* gene may contribute to disease susceptibility to POAG and NTG, but may not account for all of the phenotypic variability between individuals whose glaucoma results from variants in this gene. Our association findings suggest that the *HK2* gene that contains this polymorphism might play a role in POAG and NTG in the Japanese population, but it remains to be seen whether rs678350 is actually causative, perhaps through altering transcription or splicing, or whether another allele(s) in this gene or its regulatory region might actually be causing the disease. There remains a possibility that the p.Gln142His (A/T) SNP in *HK2* may play a role in disease pathology, but our study can only show association, not causation. Because none of our subjects come from the original families used to map the GLC1B locus, we can only draw conclusions regarding the possible role of this gene in the Japanese population, but this finding raises questions about whether this could be the GLC1B gene.

*The HK2* gene product plays an important role in intracellular glucose metabolism by catalyzing the conversion of glucose to glucose-6-phosphate. The *HK2* gene localizes to the outer membrane of mitochondria. Since reduced glucose-6-phosphate content in muscle has been demonstrated in pre-non-insulin-dependent diabetes mellitus (pre-NIDDM) and NIDDM subjects, *HK2* was investigated as a promising candidate gene for noninsulin-dependent diabetes mellitus (NIDDM; OMIM125853) [17], [18]; however, those studies concluded that mutations of the *HK2* gene, including a common p.Gln142His polymorphism is not a major etiologic factor for NIDDM in the Finnish [17], [18], [19], [20], British [19], and Danish [20] populations. In brain, mitochondrial-hexokinase activity plays a key antioxidant role protecting against oxidative stress (ROS) [21], and complements the classical antioxidant enzymes that protect against oxidative stress [22]. Hexokinase antagonizes the release of mitochondrial cytochrome C activation of Akt, which is recognized as a potent inhibitor of apoptosis. *HK2* is probably associated with an anti-oxidative reaction and inhibition of apoptosis through Bax/Bak-mediated cytochrome *c* release [23]. Leber's hereditary optic neuropathy (LHON) -associated mitochondrial DNA mutations were found in Japanese patients with POAG [24], so it is reasonable to consider a gene whose product plays a role in mitochondria as a candidate gene for other phenotypes involving optic neuropathy.

### *NCK2* genes in POAG and NTG subjects

The *NCK2* gene, which was previously reported to be associated with NTG [16], encodes a member of the NCK family of adaptor proteins, and the adaptor protein which associates with tyrosine-phosphorylated growth factor receptors of their cellular substrates. SH2/SH3 domain-containing adapter proteins, such as the NCK family, play a major role in regulating tyrosine kinase signaling [25]. Previously, microsatellite marker D2S176 within the GLC1B locus showed significant association with NTG in the Japanese population, and D2S176 is located 24 kb from the *NCK2* gene [16]. Brain-derived neurotrophic factor (BDNF) binds to and activates the TrkB tyrosine kinase receptor to regulate cell differentiation and survival in the nervous system. BDNF stimulation promotes interaction of *Nck2* with TrkB in cortical neuron [26]. And BDNF signaling in glia is known to play important roles in neural protection and regeneration, particularly in conversion of Muller glia to photoreceptors [27]. In our study, it is interesting that the *NCK2* variant rs2033008 showed a significantly difference from the control population in the NTG group, where the disease pathology seems to be focused on the retinal ganglion cells and the optic nerve, but not in the POAG group, where a substantial disease component localizes to the anterior chamber of the eye (Table 5). Thus although our study falls short of achieving a level of significance needed to identify *NCK2* de novo as a glaucoma gene and this polymorphism showed deviation from Hardy–Weinberg equilibrium (p = 0.030 in Stage One), our data do support the prior finding of significant allele frequency differences between NTG cases and normal controls in the Japanese population [16]. It is unclear whether this deviation from Hardy-Weinberg equilibrium in Stage One might represent the absence of some alleles from this population because they are associated with diagnoses specifically excluded from this study, such as ocular hypertension.

### Immunohistochemistory of the *Hk2* and *Nck2*

Although KH2 and NCK2 had previously been detected in retina, more precise localization to specific cell types is needed to begin understanding how the gene products might play a role in disease pathology. In the representative IHC photographs, antibody against *Hk2* was strongly immunoreactive in the ganglion cell layer (GCL). The *Hk2* protein localizes to the outer membrane of mitochondria, and interestingly the *Hk2* protein appears in the GCL. *Nck2*, which interacts with BDNF, is expressed in ganglion cell layer (GCL), inner nuclear layer (INL) and outer plexiform layer (OPL), and most expressed in GCL. This localization makes it highly conceivable that the *Hk2 gene products* could each play a role in glaucoma, and there is possibility that *Nck2* could have relationship with glaucoma.

### *TMEM182* in POAG and NTG

SNPs rs869833 and rs960011 in the TMEM182 gene showed significant association with POAG and NTG in Stage One that was not confirmed in the second stage. The primary amino acid sequence of *TMEM182* predicts an evolutionarily-conserved novel transmembrane protein, which consists of four putative membrane-spanning regions indicative of an integral membrane topology. The *TMEM182* protein sequence lacks homologies with previously-defined protein families. However, the pro-inflammatory cytokine TNFα down-regulates *TMEM182* transcript expression in adipocytes [28]. Its transcript expresses in white adipose tissues, heart, muscle, and lower relative levels of *TMEM182* transcript are found in kidney, testis, and brain. Identification of the intracellular signaling pathway involved in the TNFα-mediated decrease might be one clue offering insights into association between POAG and *TMEM182* function. Failure to confirm the association with POAG in the second stage could be attributable to clinical heterogeneity, but this result still needs to be confirmed in a second population. Nakano et al. demonstrated heterogeneity in the Japanese POAG population when their genome-wide association study of 1,575 Japanese POAG and normal subjects identified significant evidence of association with 6 SNPs on Chromosome 1, 10, 12 [29]. They did not report evidence of association with SNPs on Chromosome 2. Thus these SNPs on Chr2 might be the variants for which our study is not well-powered, or clinical heterogeneity might be complicating our ability to detect the association in our limited sample size.

In our data set, *HK2* shows the strongest evidence of association with NTG in the Japanese population out of all of the genes that have SNPs represented on the screening panel we used.

Even when a single simple Mendelian locus causes a disease, variants in other genes may contribute to phenotypic variability, and phenotypic complexity along with locus and allele heterogeneity can complicate the problem of identifying the underlying causes of the disease. Our findings raise questions about whether additional genes in this region may be contributing to phenotypic heterogeneity within the NTG and POAG populations. The MD values of the visual field in these studies indicate middle to advanced stages of the disease, with the range of values possibly resulting from a combination of genetic complexity and genetic heterogeneity.

On the other hand, we also have to consider the importance of apparent gene deserts. The SNP rs1239066 shows significant evidence of association in both Stage One and Stage Two screenings, and in the meta-analysis (POAG, NTG; P = 0.001, 0.005). Significant evidence was also found for rs1027003 (POAG; P = 0,010) (Table 2), but neither one is in the immediate vicinity of a known gene. Wiggs et al. found significant evidence that genetic variants in a gene desert on 8q22 are associated with optic nerve damage in glaucoma [14]. So additional follow up studies will need to explore this gene desert region to determine whether any functional sequences there are playing a role in the disease.

Glaucoma is a complex disease, and it involves genetic variants that confer moderate to low effect sizes (e.g., OR = 1.2–1.5). The OR which was identified in the first stage with the P value cutoff of 0.01 was 1.68. This cutoff value was a bit strict to exclude the false positives.

Further investigations of the structure and function of the *HK2, NCK2* and *TMEM182* proteins would be helpful in understanding the pathogenesis of POAG and NTG. Our data suggest that *HK2* may play an important role in NTG in the Japanese population; although our data suggest that *HK2* might be the GLC1B gene, a firm conclusion on the subject awaits screening of members of the families originally used to map the GLC1B locus.

## Patients and Methods

### Ethics statement

This study was approved by the Institutional Review Board of Tohoku University, Keio University, Tokyo Metropolitan Police Hospital, Niigata University, Ideta Eye Hospital, and all procedures were conducted in accordance with the Declaration of Helsinki. All participants provided written informed consent after an explanation of the purpose and procedures to publish these case details.

### Patient Recruitment and Characteristics

The samples used in the first screening (Stage One) were collected in Keio University hospitals, Tokyo Metropolitan Police Hospital, Niigata University, and Ideta Eye Hospital, and the samples used in the second screening (Stage Two) were collected at Tohoku University.

Routine ophthalmic examinations were performed on all subjects. Individuals were included as POAG cases if they fulfilled the following inclusion criteria: 1) applanation IOP greater than 22 mm Hg in each eye; 2) spherical equivalent more than −8 diopter; 3) glaucomatous cupping in each eye including cup-to-disc ratio greater than 0.7; 4) visual field loss measured by Goldmann perimetry or Humphrey automated field analyzer (Carl Zeiss Meditec, Dublin, CA) in Stage One. The severity of the visual field defects was scored from 1 to 5 according to previously reported criteria ref. The data obtained by two types of perimetry were combined using a five-point scale: 1, no alterations; 2, early defects; 3, moderate defects; 4, severe defects; and 5, light perception only or no light perception. The first four groups on this severity scale followed Kozaki's classification based on Goldmann perimetry or the classification was based on results of Humphrey automated field analyzer [30], [31]. Kozaki's classification is widely used in Japan. In Stage Two, all of the visual field loss were measured by Humphrey automated field analyzer according to Anderson-Pattela classification [30] consistent with the glaucomatous cupping in at least one eye; and 5) open anterior chamber angles; and exclusion of secondary causes (e.g., trauma, uveitis, or steroid-induced glaucoma). The criteria for NTG were the same as for POAG except that NTG subjects showed applanation IOP less than 22 mm Hg in both eyes at each examination. Baseline clinical parameters including age, gender, spherical equivalent base line visual acuity (VA), IOP measured by Goldmann applanation tonometry were recorded at the time of first diagnosis of POAG or NTG in each patient. Mean deviation (MD) values indicative of visual field damage were obtained by the Swedish interactive threshold algorithm (SITA)-standard strategy of the 30-2 program of HFA (Carl Zeiss Meditec, Dublin, Californuia, USA). MD was used on reliable visual field test results (<20% fixation errors, <33% false-positive results, and <33% false-negative results). Control subjects had these characteristics: IOP less than 22 mm Hg, normal optic discs, and no family history of glaucoma. To decrease the chance of enrolling individuals with pre-symptomatic glaucoma, we limited this group to individuals older than 60 years.

### Two Stage Screening Protocol

The two-stage screening strategy is shown in Figure 1. The first stage screened used 669 SNPs from the GLC1B region, using the Illumina panel #8 (Chromosome 2; 77–117 Mbp) (Illumina, San Diego, CA, USA) carried by Illumina (San Diego, CA, USA), and each SNP was tested for association with POAG or NTG. The first stage used a cohort of 368 unrelated Japanese individuals: 123 POAG cases (63 men and 60 women), 121 NTG cases (61 men and 60 women) and 120 normal subjects (61 men and 59 women). Mean age of POAG cases was 56.9±11.5 years. Mean age of NTG cases was 54.0±12.3 years. Mean age of controls was 70.3±10.2 years. The visual field scores were 2.8±1.0 in POAG cases and 2.7±0.9 in NTG cases (Table 9). Single-nucleotide polymorphisms (SNPs) with a call rate <90% were excluded from the analysis. All of the polymorphisms showed no deviation from Hardy–Weinberg equilibrium (*P*>0.05) except rs2033008 in Stage One (p = 0.030).

**Table 9:** Clinical Characteristics of Subjects studied in Stage One and Two Screening.

Endophenotype		Age at diagnosis (y.o.)	Maximum IOP* (mmHg)	The Visual Field Score**
Stage One	POAG	56.9±11.4	25.3±5.6	2.8±1.0
	NTG	54.0±12.2	16.0±2.3	2.7±0.9
	Control	70.3±10.2	13.9±2.2	
Stage Two	POAG	57.8±12.0	23.5±5.3	−15.0±9.0 (dB)
	NTG	56.4±13.3	16.8±2.4	−11.0±7.1 (dB)
	Control	69.7±9.3	13.9±2.2	

* IOP; intra ocular pressure (under medication).

** The Visual Field Score was evaluated by Humphrey MD value or Goldmann perimetry (Stage One) and Humphrey MD value (Stage Two).

In Stage One, the severity of the visual field defects was scored from 1 to 5 according to previously reported criteria. The data obtained by two types of perimetry were combined using a five-point scale: 1, no alterations; 2, early defects; 3, moderate defects; 4, severe defects; and 5, light perception only or no light perception. The first four groups on this severity scale followed Kozaki's classification based on Goldmann perimetry or the classification was based on results of visual field perimetry (Humphrey Field Analyzer; Carl Zeiss Meditec, Dublin, CA). Kozaki's classification is widely used in Japan.

The second stage screened SNPs that showed significant evidence for association in the first round in this study (p<0.01) and in the NCK2 gene, which previously showed association with NTG [16]. The odds ratio which was identified in the first stage with the P value cutoff of 0.01 was 1.68. Second stage screening was carried out using sequencing of DNA PCR amplified by polymerase chain reaction from genomic DNA samples from a population of 473 unrelated Japanese individuals, including 187 POAG cases (119 men and 68 women), 286 cases NTG (139 men and 147 women), and 271 control subjects (145 men and 126 women). Mean age of POAG cases was 57.8±12.0 years. Mean age of NTG cases was 56.4±13.3years. Mean age of controls was 69.7±9.3 years. Maximum intra ocular pressure under medication were 23.5±5.3 mmHg in POAG subjects and 16.8±2.4 mmHg in NTG subjects. Mean deviation (MD) value of the visual field test was −15.0±9.0 dB in POAG cases and −11.0±7.1 dB in NTG cases (Table 9).

### Sample Preparation

Genomic DNA was extracted from leukocytes of the peripheral blood. It was purified by the Qiagen QIAamp Blood Kit (Qiagen, Valencia, CA, USA).

### Mutation Screening

Mutation screening was carried out in genes that contained SNPs that showed significant evidence of association in the first stage (HK2 and TMEM182) plus the previously-reported NCK2 gene [16]. All of the exons of the *HK2*, *NCK2*, *TMSB10* and *TMEM182* genes, and positive SNPs were amplified by a polymerase chain reaction (PCR) using 0.5 µM concentration of primers in an amplification mixture (25 µl) containing 0.2 mM dNTPs and 0.5 U Ex Taq polymerase (Takara Bio, Shiga, Japan) with 30 ng template DNA. Oligonucleotides for amplification and sequencing were selected using Primer3 software (http://frodo.wi.mit.edu/cgi-bin/primer3/primer3_www.cgi/ provided in the public domain by the Massachusetts Institute of Technology, Cambridge, MA). Primers for amplification and sequencing of coding sequence were placed in introns far enough from the intron/exon junctions to allow for visualization of the splice site sequences. The PCR fragments were purified with ExoSAP-IT (USB, Cleveland, Ohio, USA), sequenced by the BigDye™ Terminator v3.1 Cycle Sequencing Kit (Perkin-Elmer, Foster City, CA, USA) on an automated DNA sequencer (ABI PRISM™ 3100 Genetic Analyzer, Perkin-Elmer, Waltham, MA, USA).

### Statistical Analysis

The significance of association was determined by contingency table analysis using Fisher's exact test or Chi-square test, depending on cell counts. In estimation of genotype-phenotype correlation, we used Dunnett's test to compare group means of those carrying the mutant variant being tested against the group means of those carrying the normal, reference sequence. Odds ratios (approximating to relative risk) were calculated as a measure of the association between the allele frequency and the phenotype of POAG/NTG, estimated using the SNPAlyze program version 7.0 (Dynacom, Yokohama, Japan). Hardy–Weinberg equilibrium was analyzed using gene frequencies obtained by simple gene counting and the chi-square test with Yates' correction for comparing observed and expected values.

### Immunohistochemistory of the *HK2* and *NCK2*

Murine retinas were fixed with 4% PFA at 4°C overnight and then cryoprotected in phosphate buffered saline (PBS) with 20% sucrose. Cryosections (thickness 10 µm) were mounted on the slides and incubated with blocking buffer (10% goat serum, 0.5% gelatin, 3% BSA and 0.2% Tween 20 in PBS). Next, they were incubated with primary antibodies at 4°C overnight. Primary antibodies used were Mouse anti-Glial Fibrillary Acidic Protein (GFAP) (1∶200; MAB360; Chemicon, Millipore, MA, USA), mouse anti-C38 (1∶200; provided by Dr. Jun Kosaka), rabbit anti-NCK2 (1∶200; ab14590; Abcam), or rabbit anti-HK2 (1∶200; 2867S; Cell Signaling Technology, MA, USA). The sections were washed three times with PBST (PBS containing 0.2% Tween 20) and then incubated with secondary goat anti-rabbit IgG antibody (1∶200; A11008 Invitrogen, Carlsbad, CA, USA) tagged with Alexa 488 or goat anti-mouse IgG A11030; Invitrogen, Carlsbad, CA, USA) tagged with Alexa 546 for 1 hour. The slides were washed three times and mounted with Vectashield mounting medium (H1000; Vector, Burlingame, CA).

## References

[1] Quigley H (1996) Number of people with glaucoma worldwide. Br J Ophthalmol 80: 389–393. 8695555 10.1136/bjo.80.5.389 PMC505485

[2] Quigley H (1993) Open-angle glaucoma. N Engl J Med 328: 1097–1106. 8455668 10.1056/NEJM199304153281507

[3] Hitchings RA, Anderton SA (1983) A comparative study of visual field defects seen in patients with low-tension glaucoma and chronic simple glaucoma. Br J Ophthalmol 67: 818–821. 6671097 10.1136/bjo.67.12.818 PMC1040211

[4] Hitchings R (1992) Low tension glaucoma–its place in modern glaucoma practice. Br J Ophthalmol 76: 494–496. 1390534 10.1136/bjo.76.8.494 PMC504325

[5] Werner E (1996) Normal-tension glaucoma.; Ritch R SM, Krupin T, eds., editor. St.Louis: Mosby. 769–797 p.

[6] Shiose Y, Kitazawa Y, Tsukahara S, Akamatsu T, Mizokami K, et al (1991) Epidemiology of glaucoma in Japan–a nationwide glaucoma survey. Jpn J Ophthalmol 35: 133–155. 1779484

[7] Iwase A, Suzuki Y, Araie M, Yamamoto T, Abe H, et al (2004) The prevalence of primary open-angle glaucoma in Japanese: the Tajimi Study. Ophthalmology 111: 1641–1648. 15350316 10.1016/j.ophtha.2004.03.029

[8] Raymond V (1997) Molecular genetics of the glaucomas: mapping of the first five “GLC” loci. Am J Hum Genet 60: 272–277. 9012399 PMC1712410

[9] Sarfarazi M (1997) Recent advances in molecular genetics of glaucomas. Hum Mol Genet 6: 1667–1677. 9300658 10.1093/hmg/6.10.1667

[10] Liu Y, Allingham RR (2011) Molecular genetics in glaucoma. Exp Eye Res 93: 331–339. 21871452 10.1016/j.exer.2011.08.007 PMC4293633

[11] Pasutto F, Keller KE, Weisschuh N, Sticht H, Samples JR, et al (2012) Variants in ASB10 are associated with open-angle glaucoma. Hum Mol Genet 21: 1336–1349. 22156576 10.1093/hmg/ddr572 PMC3284122

[12] Burdon KP, Macgregor S, Hewitt AW, Sharma S, Chidlow G, et al (2011) Genome-wide association study identifies susceptibility loci for open angle glaucoma at TMCO1 and CDKN2B-AS1. Nat Genet 43: 574–578. 21532571 10.1038/ng.824

[13] Ramdas WD, van Koolwijk LM, Lemij HG, Pasutto F, Cree AJ, et al (2011) Common genetic variants associated with open-angle glaucoma. Hum Mol Genet 20: 2464–2471. 21427129 10.1093/hmg/ddr120

[14] Wiggs JL, Yaspan BL, Hauser MA, Kang JH, Allingham RR, et al (2012) Common variants at 9p21 and 8q22 are associated with increased susceptibility to optic nerve degeneration in glaucoma. PLoS Genet 8: e1002654. 22570617 10.1371/journal.pgen.1002654 PMC3343074

[15] Stoilova D, Child A, Trifan OC, Crick RP, Coakes RL, et al (1996) Localization of a locus (GLC1B) for adult-onset primary open angle glaucoma to the 2cen-q13 region. Genomics 36: 142–150. 8812425 10.1006/geno.1996.0434

[16] Akiyama M, Yatsu K, Ota M, Katsuyama Y, Kashiwagi K, et al (2008) Microsatellite analysis of the GLC1B locus on chromosome 2 points to NCK2 as a new candidate gene for normal tension glaucoma. Br J Ophthalmol 92: 1293–1296. 18723748 10.1136/bjo.2008.139980

[17] Laakso M, Malkki M, Deeb SS (1995) Amino acid substitutions in hexokinase II among patients with NIDDM. Diabetes 44: 330–334. 7883120 10.2337/diab.44.3.330

[18] Laakso M, Malkki M, Kekalainen P, Kuusisto J, Deeb SS (1995) Polymorphisms of the human hexokinase II gene: lack of association with NIDDM and insulin resistance. Diabetologia 38: 617–622. 7489847 10.1007/BF00400733

[19] Vidal-Puig A, Printz RL, Stratton IM, Granner DK, Moller DE (1995) Analysis of the hexokinase II gene in subjects with insulin resistance and NIDDM and detection of a Gln142–>His substitution. Diabetes 44: 340–346. 7883122 10.2337/diab.44.3.340

[20] Echwald SM, Bjorbaek C, Hansen T, Clausen JO, Vestergaard H, et al (1995) Identification of four amino acid substitutions in hexokinase II and studies of relationships to NIDDM, glucose effectiveness, and insulin sensitivity. Diabetes 44: 347–353. 7883123 10.2337/diab.44.3.347

[21] da-Silva WS, Gomez-Puyou A, de Gomez-Puyou MT, Moreno-Sanchez R, De Felice FG, et al (2004) Mitochondrial bound hexokinase activity as a preventive antioxidant defense: steady-state ADP formation as a regulatory mechanism of membrane potential and reactive oxygen species generation in mitochondria. J Biol Chem 279: 39846–39855. 15247300 10.1074/jbc.M403835200

[22] Santiago AP, Chaves EA, Oliveira MF, Galina A (2008) Reactive oxygen species generation is modulated by mitochondrial kinases: correlation with mitochondrial antioxidant peroxidases in rat tissues. Biochimie 90: 1566–1577. 18634844 10.1016/j.biochi.2008.06.013

[23] Majewski N, Nogueira V, Bhaskar P, Coy PE, Skeen JE, et al (2004) Hexokinase-mitochondria interaction mediated by Akt is required to inhibit apoptosis in the presence or absence of Bax and Bak. Mol Cell 16: 819–830. 15574336 10.1016/j.molcel.2004.11.014

[24] Inagaki Y, Mashima Y, Fuse N, Ohtake Y, Fujimaki T, et al (2006) Mitochondrial DNA mutations with Leber's hereditary optic neuropathy in Japanese patients with open-angle glaucoma. Jpn J Ophthalmol 50: 128–134. 16604388 10.1007/s10384-005-0290-0

[25] Buday L, Wunderlich L, Tamas P (2002) The Nck family of adapter proteins: regulators of actin cytoskeleton. Cell Signal 14: 723–731. 12034353 10.1016/s0898-6568(02)00027-x

[26] Suzuki S, Mizutani M, Suzuki K, Yamada M, Kojima M, et al (2002) Brain-derived neurotrophic factor promotes interaction of the Nck2 adaptor protein with the TrkB tyrosine kinase receptor. Biochem Biophys Res Commun 294: 1087–1092. 12074588 10.1016/S0006-291X(02)00606-X

[27] Harada C, Guo X, Namekata K, Kimura A, Nakamura K, et al (2011) Glia- and neuron-specific functions of TrkB signalling during retinal degeneration and regeneration. Nat Commun 2: 189. 21304518 10.1038/ncomms1190 PMC3105320

[28] Wu Y, Smas CM (2008) Expression and regulation of transcript for the novel transmembrane protein Tmem182 in the adipocyte and muscle lineage. BMC Res Notes 1: 85. 18803820 10.1186/1756-0500-1-85 PMC2564950

[29] Nakano M, Ikeda Y, Taniguchi T, Yagi T, Fuwa M, et al (2009) Three susceptible loci associated with primary open-angle glaucoma identified by genome-wide association study in a Japanese population. Proc Natl Acad Sci U S A 106: 12838–12842. 19625618 10.1073/pnas.0906397106 PMC2722348

[30] Anderson DR, VM P (1999) Automated Static Perimetry. 2nd edition. St.Louis: Mosby.

[31] Funayama T, Ishikawa K, Ohtake Y, Tanino T, Kurosaka D, et al (2004) Variants in optineurin gene and their association with tumor necrosis factor-alpha polymorphisms in Japanese patients with glaucoma. Invest Ophthalmol Vis Sci 45: 4359–4367. 15557444 10.1167/iovs.03-1403

